# Viral diversity in wild rodents in the regions of Canaã de Carajás and Curionopólis, State of Pará, Brazil

**DOI:** 10.3389/fmicb.2024.1502462

**Published:** 2025-01-07

**Authors:** Adriana Freitas Moraes Monteiro, Fábio Silva da Silva, Ana Cecília Ribeiro Cruz, Sandro Patroca da Silva, Alice Louize Nunes Queiroz, Livia Medeiros Neves Casseb, Livia Carício Martins, Daniele Barbosa de Almeida Medeiros

**Affiliations:** ^1^Graduate Program in Virology, Evandro Chagas Institute — IEC/MS/SVSA, Ananindeua, Brazil; ^2^Evandro Chagas Institute — IEC/MS/SVSA, Department of Arbovirology and Hemorragic Fevers, Ananindeua, Brazil

**Keywords:** *Arenaviridae*, epidemiologic vigilance, *Filoviridae*, metagenomics, rodent

## Abstract

Wild rodents serve as crucial reservoirs for zoonotic viruses. Anthropogenic and environmental disruptions, particularly those induced by mining activities, can destabilize rodent populations and facilitate the emergence of viral agents. In the Canaã dos Carajás and Curionópolis regions of Brazil, significant environmental changes have occurred due to mining expansion, potentially creating conditions conducive to the emergence of rodent-associated viral diseases. This study aimed to investigate the viral diversity in wild rodents captured in Canaã dos Carajás and Curionópolis, Pará, between 2017 and 2019. A total of 102 rodent samples were taxonomically identified through karyotyping and screened for anti-*Orthohantavirus* antibodies using the ELISA method. Subsequently, nucleotide sequencing and bioinformatics analyses were conducted on 14 selected samples to characterize the virome. This selection was based on the most commonly associated rodent genera as reservoirs of *Orthohantavirus* and *Mammarenavirus*. Of the 102 samples tested via ELISA, 100 were negative, and two showed optical density at the cutoff point. Sequencing of the 14 samples generated approximately 520 million reads, with 409 million retained after quality control. These reads were categorized into 53 viral families, including both DNA and RNA viruses, with *Retroviridae, Baculoviridae*, and *Microviridae* being the most abundant. Viral contigs were identified, including one fragment related to *Arenaviridae* and three to *Filoviridae*. Metagenomic analysis revealed high viral diversity in the sampled rodents, with the presence of viral families of public health concern, such as *Arenaviridae* and *Filoviridae*. The findings suggest that increased human activities associated with mining may contribute to the emergence of these viruses, underscoring the need for ongoing surveillance to prevent potential outbreaks.

## 1 Introduction

Emerging infectious diseases, particularly those of zoonotic origin, have become a significant public health challenge in recent decades (Rahman et al., 2020). Among animals, rodents play a crucial role as reservoirs for many viruses due to their species diversity, varied habitats, and tendency to live close to human-inhabited areas (Luis et al., 2013; Han et al., 2015; Carlson et al., 2019). Viral diversity in rodents can be influenced by anthropogenic factors such as deforestation, changes in land use, and climate change (Aguirre, 2017; Plowright et al., 2021; Rupasinghe et al., 2022), which can disrupt ecological relationships, alter host dynamics, and promote the increase and/or emergence of viral agents (Thompson, 2013; Horefti, 2023).

Advances in next-generation sequencing (NGS) technologies combined with bioinformatics have revealed a vast viral diversity among rodent populations, whether or not they are associated with human diseases (Yin et al., 2022; Zhao et al., 2022). Among the most commonly described viruses in these reservoirs are hantaviruses and arenaviruses (Families *Hantaviridae* and *Arenaviridae*), known to cause serious diseases in humans, such as *Hantavirus pulmonary syndrome* (HPS) and hemorrhagic fevers. However, other viral families have also been identified, including *Astroviridae* (Phan et al., 2011; Yin et al., 2022), *Togaviridae, Reoviridae* (He et al., 2022), *Coronaviridae* (Tsoleridis and Ball, 2020; Wang et al., 2020), *Flaviviridae* (Meerburg et al., 2009; Han et al., 2015; Tirera et al., 2021), *Arteriviridae, Anelloviridae*, and *Circoviridae*, among others (Wu et al., 2021; He et al., 2022). These discoveries are crucial for the identification and surveillance of viral pathogens as well as for the prevention of potential outbreaks (Wu et al., 2016).

In Brazil, regions such as Canaã dos Carajás and Curionópolis, in the southwest of the State of Pará, are notable for their rich biodiversity and significant mineral reserves. However, these areas have faced intense environmental impacts due to deforestation, largely driven by the expansion of mining projects, road construction, agricultural growth, and the emergence of small villages. These environmental changes create favorable conditions for the emergence and spread of viral diseases associated with rodents (Ferreira et al., 2005).

In this study, we used a metagenomic approach to characterize viral diversity in wild rodents from the regions of Canaã dos Carajás and Curionópolis. Our objective is to investigate the viral composition in these animals and contribute to a better understanding of the viral dynamics in these reservoirs and their potential risks to public health.

## 2 Materials and methods

### 2.1 Rodent samples

**Figure 1:**
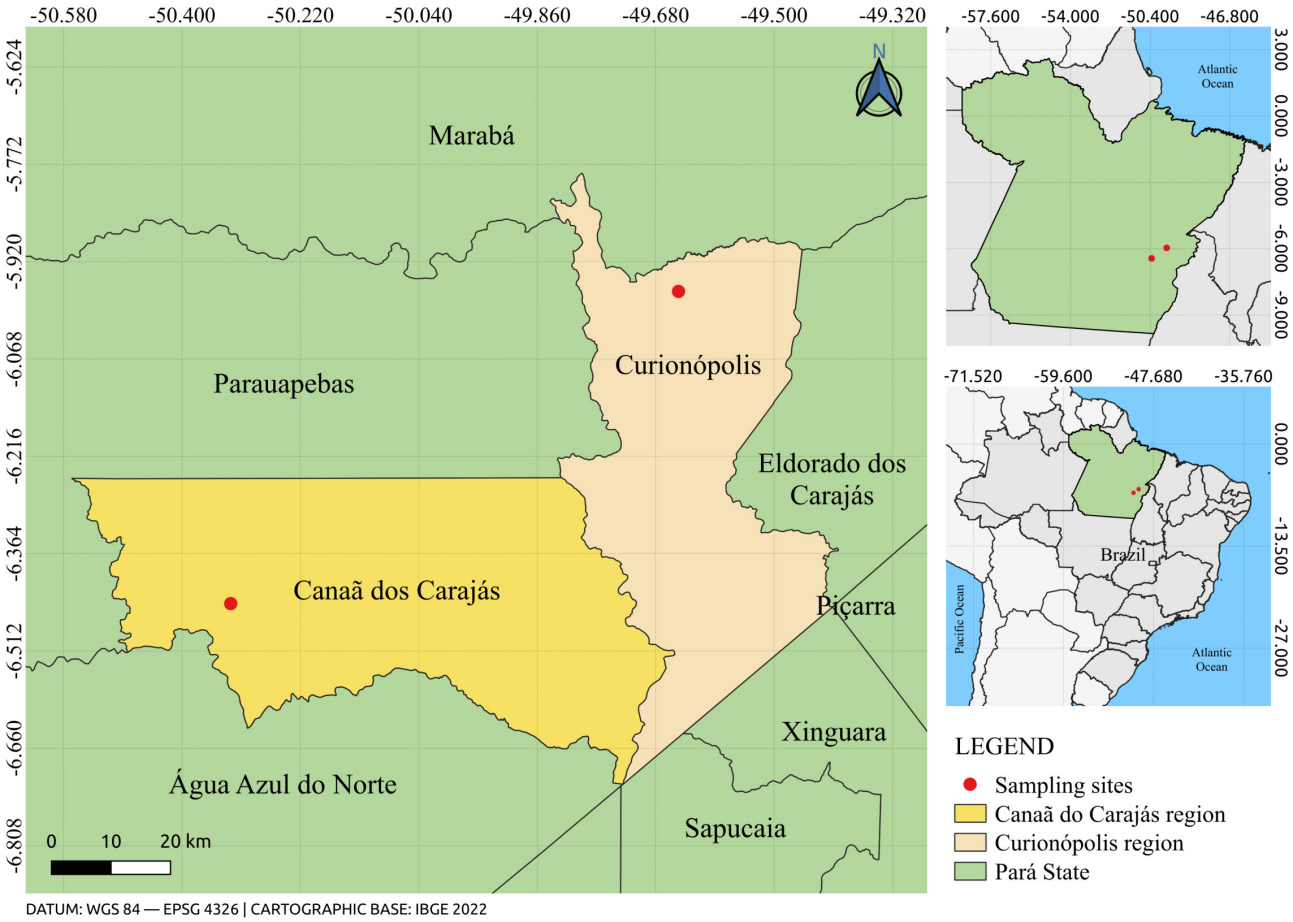
Map showing the location of rodent capture sites in the municipalities of Canaã dos Carajás and Curionópolis, State of Pará, Brazil. The figure was created using QGIS v.3.10.4 software (available at https://qgis.org/pt_BR/site), in conjunction with the IBGE 2022 database (available at: https://www.ibge.gov.br/).

To conduct this study, 102 biological samples of wild rodents were used, captured during eco-epidemiological expeditions in the municipalities of Canaã dos Carajás and Curionópolis (Figure 1), located in the State of Pará, between 2017 and 2019. All samples are stored in the Arbovirology Section of the Evandro Chagas Institute. The rodent samples were identified using the code RO, followed by a specific number, designated as registered identification. This code served as a unique reference for each sample throughout the analysis process, ensuring the traceability and organization of the data obtained during the study. The taxonomic identification of the captured rodents was performed by means of karyotyping (Bovincino et al., 2008).

### 2.2 Enzyme-linked immunosorbent assay for detection of anti-orthohantavirus antibodies

The ELISA was conducted as a screening method using 102 selected rodent samples. For this purpose, a truncated recombinant protein, HTN-120, based on the N gene of orthohantaviruses circulating in the Amazon and developed by the Evandro Chagas Institute, was used. ELISA microplates were coated with 4 μg of the recombinant HTN-120 antigen and a negative antigen control, then incubated overnight at 4°C. The plates were washed four times with PBS-Tween buffer, blocked with PBS-Tween and lectin solution, and incubated for 1 h at 37°C. After blocking, rodent sera/blood samples were added at 1:100 and 1:400 dilutions and incubated for an additional hour at 37°C. The plates were washed again, and an anti-*Peromyscus* IgG conjugate, diluted 1:500, was added and incubated for 1 h at 37°C. The plates were then washed and developed with TMB One Solution for 15 min at 37°C. The reaction was stopped with 1M HCl, and the absorbance was read at 450 nm. Samples with an optical density (OD) below 0.2 were considered negative.

### 2.3 RNA extraction

For RNA extraction, fragments of viscera (brain, lung, kidney, spleen, and heart) were used, with pools formed for each species of captured rodent. After preparing the pools, 1 mL of Trizol RNA isolation reagent was added for every 10 mg of tissue per microtube, along with a 5 mm diameter stainless steel sphere. The samples were macerated in the Tyssueliser II (Qiagen) equipment for 2 min at a frequency of 25 Hz in the level 3 biosafety laboratory (NB3) of the Arbovirology section of the Evandro Chagas Institute. Immediately afterward, the lysed content was transferred to a tube containing a phase separation polymer (phasemaker) and incubated for 5 min at room temperature (RT). Next, 200 μL of chloroform was added, and the microtubes were vortexed for 15 s and incubated for 15 min at RT. Subsequently, the samples were centrifuged in a refrigerated centrifuge (4°C) for 5 min at 12,000 xg. The aqueous phase was removed and transferred to a new tube containing an equal volume of 70% ethanol and homogenized by vortexing for 15 s.

For purification, the PureLink RNA Mini kit (Invitrogen) was used. The aqueous solution was transferred to the column and centrifuged at 12,000 × g for 15 s. Washes were performed with wash buffers I and II, followed by another centrifugation at 12,000 × g for 15 s. After this process, the samples were eluted with 60 μL of ultrapure water (Invitrogen), incubated for 1 min at RT, and centrifuged again at 12,000 × g for 2 min. Finally, the extracted RNA was quantified using the Qubit 4.0 equipment (Invitrogen) with the Qubit RNA HS assay kit (Invitrogen), following the manufacturer's recommendations, and stored in a freezer at −70°C.

### 2.4 Double-stranded cDNA synthesis, genomic library construction and sequencing

The preparation of cDNA from RNA began with the synthesis of the first and second cDNA strands, using the SuperScript™ IV VILO™ Master Mix (Invitrogen) and NEBNext mRNA Second Strand Synthesis Module (New England BioLabs), respectively. The cDNA purification was performed using the PureLink^®^ PCR Purification Kit (Invitrogen). The synthesized cDNA was then quantified using the Assay DNA HS Kit (Invitrogen) on the Qubit 4.0 equipment (Invitrogen). The genomic library was constructed according to the Nextera XT DNA kit (Illumina) protocol guidelines and sequenced using the NextSeq 500 platform (Illumina, Inc) with the NextSeq 500/550 High Output Kit v2.5 (Illumina) in a 300-cycle reaction (paired-end, 2 × 150), following the manufacturer's recommendations.

### 2.5 Computational analysis

The files obtained after sequencing were initially subjected to quality assessment using Fastp v.0.23.4 (Chen et al., 2018). This tool was configured to remove adapter sequences and reads with a Phred quality score < 20 and a length < 50 nt. Additional filtering of ribosomal reads was performed using SortMeRNA v.2.1 (Kopylova et al., 2012) with the default sequence database provided by the tool.

The assessment of potential viral read diversity, targeting the Family taxonomic level, was performed using DIAMOND tool v.2.1.9.163 (Buchfink et al., 2021) (Blastx, e-value of 10^−5^, taxonlist 10239) with a non-redundant protein database (NR—NCBI, available at https://ftp.ncbi.nlm.nih.gov/blast/db/FASTA/). The results were compiled into table files to calculate general metrics of Alpha diversity (abundance heatmaps and diversity indices) and Beta diversity (Bray-Curtis dissimilarity matrix and PCoA), using the R language along with the pheatmap, vegan, and ggplot2 libraries.

After quality control, the data were subjected to genomic assembly using the *de novo* method with MEGAHIT software v.1.2.9 (Li et al., 2015) in its default configuration (*k*-mers ranging from 21 to 141 nt). The assembled contigs were then compared with a non-redundant protein database (NR—NCBI), targeting viral sequences using DIAMOND tool v.2.1.9.163 (Blastx, e-value of 10^−5^, taxonlist 10239), followed by manual inspection with Geneious software v.11.1.5 (Kearse et al., 2012). Viral contigs were identified and annotated based on homology searches in databases such as NCBI (using the BlastX tool) and EMBL (using the InterProScan tool to verify functional domains). Additionally, confirmation of host species, initially identified based on morphological aspects, was verified through the analysis of mitochondrial contigs, with a particular focus on the Cytochrome B (*CytB*) subunit, considering a species confirmation threshold of 98%. Functional annotation of the obtained mtDNAs was performed using the Mitochondrial Genome Annotation Server (MITOS) (Bernt et al., 2013) (available at https://usegalaxy.eu/).

The reconstruction of the phylogenies of the identified viruses was performed by considering their respective contigs along with other sequences from their respective groups available in the NCBI database. The sequences were initially aligned using the MAFFT v.7.520 algorithm (Katoh et al., 2002). The best amino acid and nucleotide substitution models were determined, followed by phylogeny reconstruction using the Maximum Likelihood method with bootstrapping values (BPP) set to 1,000 replicates, performed using IQtree v.1.6.12 (Nguyen et al., 2015). Finally, the obtained topologies were visualized using Figtree v.1.4.496 (available at http://tree.bio.ed.ac.uk/software/figtree/) and edited using Inkscape v.0.92 (available at https://inkscape.org/pt-br/).

## 3 Results

### 3.1 Enzymatic immunoassay for detection of anti-*Orthohantavirus* antibodies

The 102 rodents collected and considered for this study were taxonomically identified in nine genera, as shown in Supplementary Table 1. Due to the observation of very similar morphological and chromosomal characteristics, some species of rodents collected were identified only to the taxonomic level of genus. Samples from all 102 collected specimens were tested by ELISA for detection of IgG antibodies to *Orthohantavirus*. Of these, 100 presented OD lower than 0.2, and therefore were considered negative, while two samples presented OD at the cut-off: 0.239 for a rodent captured in Canaã dos Carajás (*Oligoryzomys microtis*, sample RO23088) and 0.261 for another captured in Curionópolis (*Necromys lasiurus*, sample RO23100).

### 3.2 Samples submitted for metagenomic analysis

The selection of samples for metagenomic study was carried out based on the rodent genera already described as potential reservoirs of *Orthohantavirus* and *Mammarenavirus*: *Proechimys* (2), *Oligoryzomys* (3), *Oryzomys* (1), *Necromys* (2), *Calomys* (1), *Oxymycterus* (2) and *Oecomys* (1) (Levis et al., 1998; Johnson et al., 1999; Moncayo et al., 2001; Travassos da Rosa et al., 2005; Milazzo et al., 2010; Oliveira et al., 2011; Travassos da Rosa et al., 2011; Firth et al., 2012; Travassos da Rosa et al., 2012; Oliveira et al., 2014; Nunes et al., 2015; Tapia-Ramírez et al., 2022). Rodents from different genera were selected to expand the knowledge about their viral diversities. Furthermore, among the rodents selected for metagenomic study, some were identified, taxonomically, only up to the genus level, with their identification at the species level being confirmed through the characterization of mtDNA (*CytB* subunit). Thus, a total of ~520 million reads were generated from genomic sequencing. Of these, 409 million reads (78.6%) remained after quality control, as detailed in Supplementary Table 2, together with the identification and confirmation of rodent species based on the *CytB* subunit.

### 3.3 Diversity analysis

**Figure 2:**
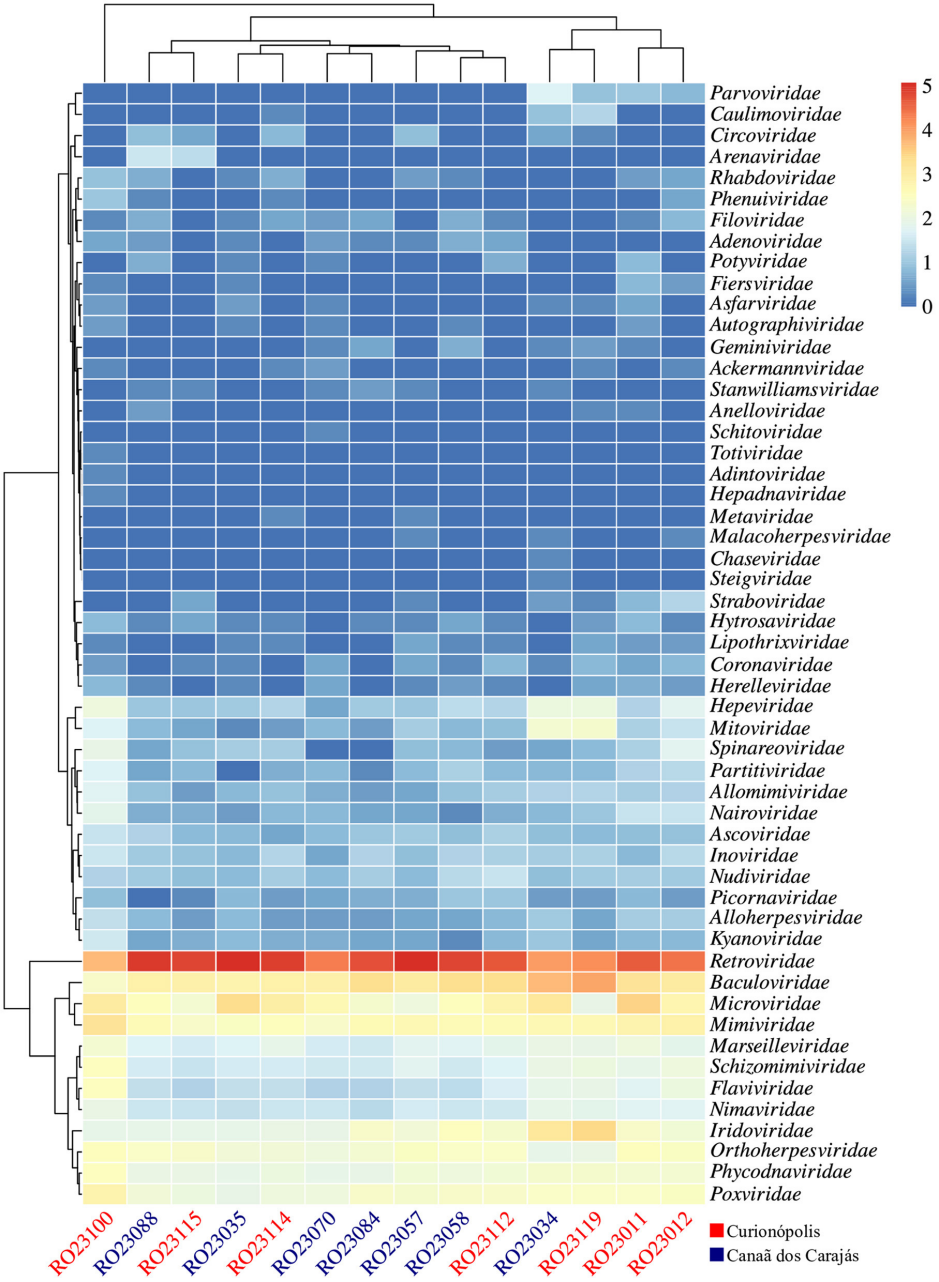
Heatmap of viral reads, normalized on a log_10_ scale, showing 53 viral families detected across the evaluated samples. The clusters indicate groupings based on abundance levels. The samples are displayed on the *x*-axis, and the potentially detected viral families are displayed on the *y*-axis. Samples from the regions of Canaã dos Carajás and Curionópolis are highlighted in blue and red, respectively.

Based on the analysis of the diversity of viral readings obtained, from the comparison with the reference database considered, it was possible to identify a wide variety of viruses, both DNA and RNA, potentially present in the evaluated samples, which were distributed in 53 families, with the most abundant being *Retroviridae, Baculoviridae* and *Microviridae* (Figure 2; Supplementary Table 3), corresponding to approximately 96.7% of the classified readings.

Samples RO23035 (*Oecomys bicolor*) and RO23057 (*Oryzomys megacephalus*) exhibited the highest abundance of classified viral reads, particularly related to the *Retroviridae* family, while sample RO23100 (*N. lasiurus*) had the lowest abundance index (Supplementary Figure 1A). However, when evaluating the diversity indices, particularly the richness of viral families, sample RO23100 showed the highest records, as indicated by the Shannon and Simpson indices. This result reflects a greater equitability (distribution of reads among represented viral families) in this sample. Despite being collected from different locations, the metagenomic profiles of the evaluated samples showed relative similarity, grouping them according to the diversity indices and as demonstrated by the principal component plot (PCoA) based on the Bray-Curtis dissimilarity matrix (Supplementary Figures 1B, 2).

### 3.4 Potential viral contigs identified

#### 3.4.1 Arenaviridae

**Figure 3:**
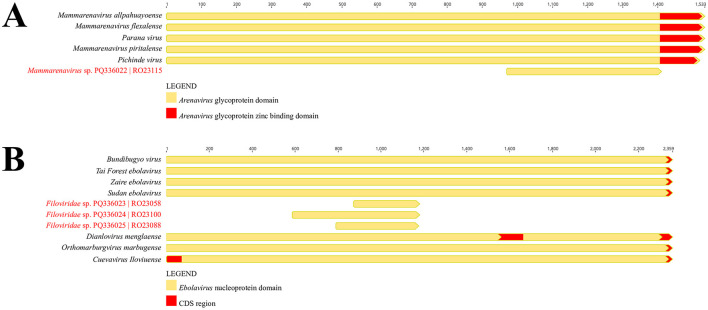
**(A)** Representation of the functional domains of the *Arenaviridae* glycoprotein region, comparing the identified contig with other members of the viral family. **(B)** Representation of the functional domains of the *Filoviridae* nucleoprotein region, comparing the identified contigs with other members of the viral family (BioProject PRJNA1157879).

A viral fragment related to the *Arenaviridae* family was obtained from a rodent sample of the species *O. microtis* (sample RO23115), captured during ecoepidemiological expeditions in the municipality of Curionópolis. The identified contig was 441 nt long and included functional domains associated with the glycoprotein region of *Arenaviridae* (Figure 3A), sharing an amino acid identity of 64% with *Mammarenavirus piritalense*.

**Figure 4:**
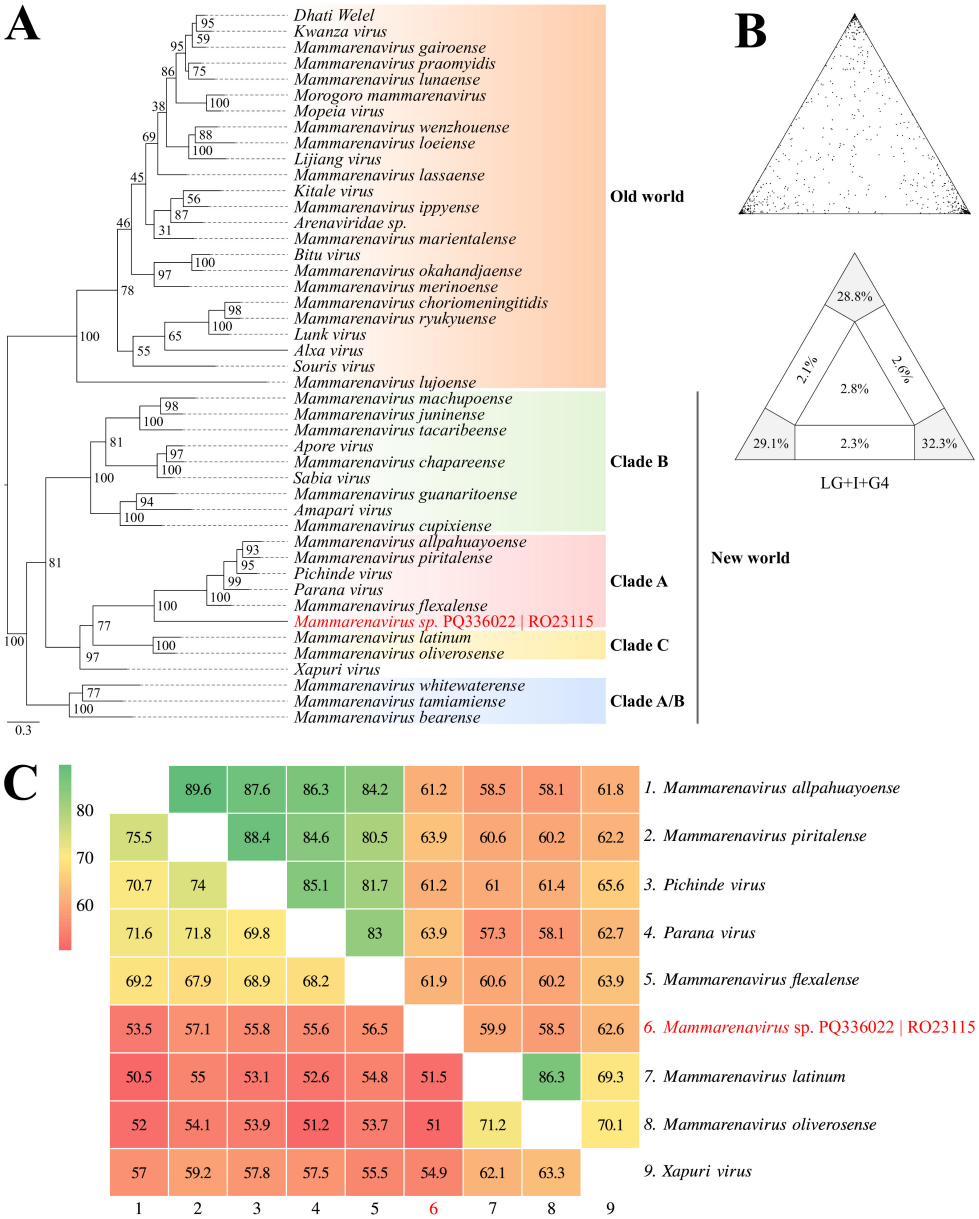
**(A)** Phylogenetic reconstruction using the Maximum Likelihood method based on amino acid sequences from the glycoprotein region of New and Old World Arenaviruses, including the identified contig highlighted in red. **(B)** Maximum likelihood mapping diagram showing the quality of the phylogenetic signal from the quartet analysis, with 90.2% of quartets resolved. **(C)** Heatmap of nucleotide (lower triangle) and amino acid (upper triangle) identities between representatives of clades **(A, C)** of New World Arenaviridae, including the identified contig (BioProject PRJNA1157879).

Based on the phylogenetic reconstruction using the Maximum Likelihood method, incorporating amino acid and nucleotide information from a set of 45 *Arenaviridae* sequences representing New and Old World species, it was observed that the identified contig clustered with taxa belonging to clade A of New World Arenaviruses. This clustering was supported by 90.2% (LG+I+G4 substitution model) and 81.6% (GTR+F++R6 substitution model) of quartets resolved during the reconstruction of the amino acid and nucleotide phylogenies, respectively (Figures 4A, B; Supplementary Figure 3). The nucleotide and amino acid identity percentages, particularly between representatives of clades A and C of *Arenaviridae* in the New World, are shown in Figure 4C.

#### 3.4.2. Filoviridae

Three viral contigs potentially associated with the *Filoviridae* family were identified from rodent samples of the species *N. lasiurus* (samples RO23058 and RO23100) and *O. microtis* (sample RO23088), identified based on the *CytB* gene. Of these samples, two were captured in the Canaã dos Carajás region (RO23058 and RO23088), and one was from the Curionópolis region (RO23100). The contigs recovered from rodents captured in Canaã dos Carajás were 303 nt (RO23058) and 393 nt (RO23088) long, featuring functional domains associated with the *Filoviridae* nucleoprotein region, and shared amino acid identities of 61.8 and 60.7% with *Bundibugyo virus* and *Zaire Ebolavirus*, respectively (Figure 3B). Similarly, the contig from sample RO23100, collected in Curionópolis, was 588 nt long, also associated with the *Filoviridae* nucleoprotein region, and shared an amino acid identity of 53.8% with *Bundibugyo virus* (Figure 3B).

**Figure 5:**
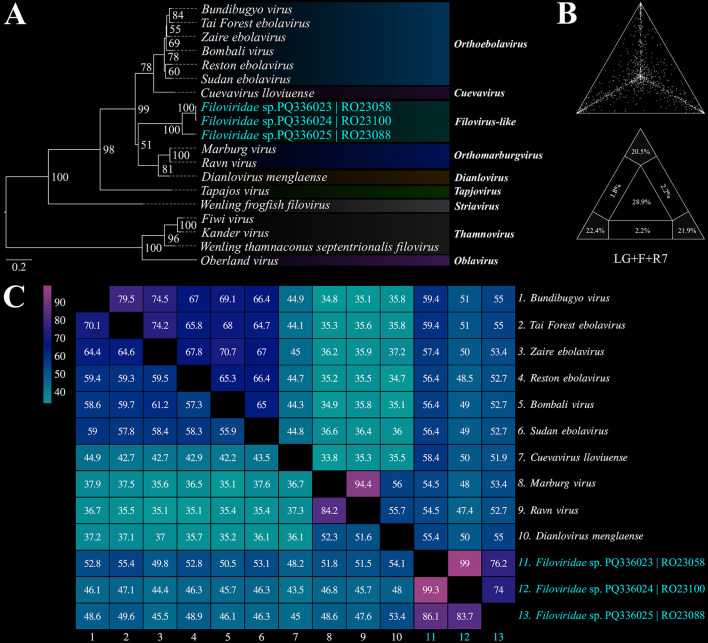
**(A)** Phylogenetic reconstruction using the Maximum Likelihood method based on amino acid sequences of *Filoviridae*. **(B)** Maximum Likelihood mapping diagram showing the quality of the phylogenetic signal from the quartet analysis, with 64.8% of quartets resolved. **(C)** Heatmap of nucleotide (lower triangle) and amino acid (upper triangle) identities between the identified contigs and representatives of the genera *Orthoebolavirus, Cuevavirus, Orthomarbugvirus*, and *Dianlovirus* (BioProject PRJNA1157879).

Based on the phylogenetic reconstruction using the Maximum Likelihood method, incorporating amino acid and nucleotide information from a set of 16 *Filoviridae* family representatives, it was observed that while the identified contigs were positioned in the same general region of the phylogenetic tree, their exact placement varied depending on the sequence model analyzed. When using the amino acid alignment, the contigs clustered closely with the clade comprising taxa from the genera *Orthomarbugvirus* and *Dianlovirus* (Figures 5A, B). Conversely, when evaluated using nucleotide alignment, the contigs were associated as a sister group to the clade containing *Orthoebolavirus* and *Cuevavirus* (Supplementary Figure 4). Despite the variable positioning, the contigs, along with the cited genera, formed a monophyletic and well-supported group (BS_amino_ = 99% and BS_nucleo_ = 93%). Although the topologies align with current molecular taxonomic classifications for this viral group, the number of unresolved quartets in each phylogenetic analysis approached or exceeded the 30% threshold, underscoring the need for a larger sample set to accurately define the observed groupings. Additionally, the percentages of nucleotide and amino acid identity between the potential *Filoviridae* viral fragments and representatives of the genera *Orthoebolavirus, Cuevavirus, Orthomarbugvirus*, and *Dianlovirus* are shown in Figure 5C.

## 4 Discussion

The municipalities of Canaã dos Carajás and Curionópolis, located in the southwest of the state of Pará, Brazil, are known for their rich biodiversity of Amazonian flora and fauna (Palheta et al., 2015). These areas are also recognized for their mineral reserves, including copper, iron, and nickel (Dos Santos et al., 2018). However, intense mining activities in these regions can lead to habitat destruction for various animals, including rodents (Furtado et al., 2023). This study utilized metagenomic analysis to explore the rodent virome and enhance our understanding of the viral diversity present in these regions.

Initially, 102 rodent samples were used to develop the study. These samples were screened by ELISA as a strategic way to verify whether any of these rodents had anti-*Orthohantavirus* antibodies and to direct the selection of samples for metagenomic study. Thus, of the 102 rodent samples, 100 presented OD values below 0.2, being considered negative, while two samples were defined as indeterminate, presenting OD at the cutoff point: one from *O. microtis* (sample RO23088), captured in Canaã dos Carajás, and another from *N. lasiurus* (sample RO23100), captured in Curionópolis.

In Brazil, the rodent *O. microtis* has been described as a reservoir of the *Rio Mamoré orthohantavirus* (RIOMV) in the states of Amazonas (Santos et al., 2006) and Acre (Nunes et al., 2015), while the rodent *N. lasiurus* was associated as the main reservoir of the *Araraquara virus* (ARAV) in the states of São Paulo, Minas Gerais, Mato Grosso do Sul, and Paraná (Suzuki et al., 2004; Santos et al., 2006; Raboni et al., 2009; Oliveira et al., 2011; dos Santos et al., 2011; Travassos da Rosa et al., 2012). In the state of Pará, in the municipality of Altamira, human cases of Hantavirus Cardiopulmonary Syndrome (HCPS) were associated with the *Castelo dos Sonhos virus* (CASV) (Johnson et al., 1999). Subsequently, studies carried out in Campo Novo do Parecis, in the state of Mato Grosso, identified the rodent *Oligorizomys utiaritensis* as the reservoir of CASV (Travassos da Rosa et al., 2011).

Later, ~520 million reads were identified in the metagenomic profile, with 409 million remaining after quality control. These reads were assigned to 53 distinct viral families. Notably, around 43.75% of these reads were related to 14 families that infect vertebrates, such as *Arenaviridae, Coronaviridae, Filoviridae, Flaviviridae, Picornaviridae*, and *Retroviridae*. These families are particularly relevant to public health, as many of their members can cause diseases in humans and other vertebrate animals. Furthermore, 46.88% of the reads were associated with 15 viral families infecting invertebrates (e.g., *Ascoviridae, Baculoviridae, Iridoviridae*), plants (e.g., *Caulimoviridae, Potyviridae*), and bacteria (e.g., *Myoviridae, Siphoviridae*). The remaining 9.38% of the reads were linked to other hosts, including fungi (e.g., *Partitiviridae*) and amoebas (e.g., *Marseilleviridae*). These results underscore the capacity of these hosts to harbor a wide variety of viruses.

Analysis of the alpha and beta diversity indices revealed that samples RO23035 (*O. microtis*) and RO23057 (*Oryzomys* sp.), collected from rodents in Canaã dos Carajás, had the highest abundance of viral reads, predominantly associated with the *Retroviridae* family. Conversely, sample RO23100 (*N. lasiurus*), from a rodent captured in Curionópolis, showed the lowest abundance of viral reads but exhibited the highest diversity index, indicating a greater richness of viral families. This finding was supported by the Shannon and Simpson indices, which demonstrated a better distribution of viral families in sample RO23100. Despite this high diversity index, the metagenomic profiles of samples from the two regions showed similarities, with abundant reads for the families *Baculoviridae, Iridoviridae, Marseilleviridae, Microviridae, Mimiviridae, Phycodnaviridae, Poxviridae*, and *Retroviridae*. This distribution may relate to the feeding habits of some rodent species (e.g., consumption of plants and insects), environmental contamination of habitats, or changes in the ecological dynamics due to human activities in these areas (Zhao et al., 2022).

Kane et al. (2024) observed that viral diversity in rodents is positively correlated with habitat disturbance. Their studies revealed that rodents such as *Rattus tanezumi*, captured in homes and agricultural fields, exhibited high viral diversity in their metagenomic profiles. Conversely, *Rattus andamanensis* found in rubber tree plantations had higher viral diversity compared to individuals of the same species from less disturbed tropical forests. These findings suggest that increased habitat disturbance due to human activities may lead to greater virome diversity in rodents, highlighting the impact of environmental changes on viral composition.

In this study, metagenomic analysis identified four virus-like fragments: one associated with the *Arenaviridae* family and three with the *Filoviridae* family. The *Arenaviridae* family consists of ambisense RNA viruses with genomes approximately 10.5 kb in size. These viruses infect mammals (mammarenaviruses), snakes (hartmaniviruses and reptarenaviruses), and fish (antennaviruses) (Radoshitzky et al., 2019).

The genome of *Arenaviridae* is divided into two main segments: the L (large) segment, encoding the RNA-dependent RNA polymerase (RdRP) and the matrix protein (Z), and the S (small) segment, encoding the nucleoprotein (NP) and the glycoprotein (GP) (Radoshitzky et al., 2023). Among the recovered fragments, one associated with the *Arenaviridae* family was identified from a rodent of the genus *Oligoryzomys* (sample RO23115), captured in the Curionópolis region. This fragment contained functional domains within the glycoprotein region and shared 64% amino acid identity with *Mammarenavirus piritalense*, a virus discovered in Venezuela associated with the rodent *Sigmodon alstoni* (Fulhorst et al., 1997). Additionally, the rodent *O. microtis* has been described as a reservoir for *Chapare virus* (CHPV), which causes Bolivian hemorrhagic fever (Loayza et al., 2022). These findings suggest possible circulation of arenaviruses in rodents in the Curionópolis region, similar to viruses known to infect humans in other parts of South America. However, further sampling is needed to enhance the robustness of these results (Fulhorst et al., 1997; Loayza et al., 2022).

In Brazil, the natural reservoir for the Brazilian *Mammarenavirus* (*Sabiá virus*), which causes Brazilian hemorrhagic fever, remains unidentified (Nastri et al., 2022). It is suspected that a rodent species may be the host, underscoring the importance of identifying potential natural hosts for epidemiological surveillance and reinforcing the significance of metagenomic studies (Mello et al., 2020; Lendino et al., 2024).

The *Filoviridae* family comprises negative-sense, linear, non-segmented RNA viruses, ~13.1 to 20.9 kb in size, that infect fish, mammals, and reptiles. This family contains five open reading frames (ORFs) encoding a nucleoprotein (NP), a polymerase cofactor (VP35), a glycoprotein (GP), a transcriptional activator (VP30), and a large protein (L) with an RNA-directed RNA polymerase (RdRP) domain (Biedenkopf et al., 2023). It is divided into six genera: *Cuevavirus, Dianlovirus, Orthoebolavirus, Orthomarburgvirus, Striavirus*, and *Thamnovirus* (Kuhn et al., 2019). The genera *Orthoebolavirus* and *Orthomarburgvirus* include viruses known to cause human diseases, such as *Ebola virus* (EBOV), *Sudan virus* (SUDV), *Bundibugyo virus* (BDBV), *Tai Forest virus* (TAFV), *Reston virus* (RESTV), *Marburg virus* (MARV), and *Ravn virus* (RAVV). Other viruses like *Bombali virus* (BOMV) and *Lloviu virus* (LLOV) (genus *Cuevavirus*) and *Mêngla virus* (MLAV) (genus *Dianlovirus*) are known members of this family but have not been associated with human diseases.

Peterson et al. (2007) propose that some rodent groups might act as potential reservoirs for filoviruses due to their wide geographic distribution, which overlaps with known areas of Ebola and Marburg outbreaks in Africa. However, they stress the necessity for further research, particularly in *filovirus-endemic* regions, to confirm this hypothesis.

Taylor et al. (2010, 2011) have highlighted a significant evolutionary association between rodents and filoviruses. Their studies revealed the presence of integrated non-retroviral viral elements (NIRVs) in rodent genomes, which are similar to the nucleoprotein (NP) and polymerase cofactor (VP35) proteins of filoviruses. In 2010, they identified these NIRVs in mice and rats, suggesting an ancient coevolution that may affect the biological functions of these elements, potentially related to immune responses. In 2011, they confirmed the conservation of these genes in rodents over millions of years, indicating that these elements might have been co-opted for essential biological functions rather than being mere viral remnants. Although the absence of live viruses or antibodies in rodents raises questions about their role as current reservoirs, the persistence of these NIRVs points to a long-standing evolutionary interaction between filoviruses and rodents, with possible implications for the ecology and evolution of filoviruses.

Recent research by Taylor and Barnhart (2023) identified an orthologous *Filovirus-like* nucleoprotein in murid and spalacid rodents. Structural and phylogenetic analyses suggest that these integrated elements may serve important biological functions, preserved for over 20 million years, possibly through purifying selection. This evidence supports a long history of interaction between rodents and filoviruses, indicating that these animals may have been ancient reservoirs. Additionally, the relationship between NIRVs in Australian marsupials and African filoviruses suggests that regions of the New World may harbor previously undetected filoviruses or could be sources for existing filoviruses.

Dupuy et al. (2023) emphasize the need to investigate potential natural reservoirs for filoviruses, including rodents, despite most current evidence focusing on bats. Their study underscores the importance of further research to clarify the role of rodents in *Filovirus* ecology, particularly in regions where the coevolution of these viruses with their hosts remains poorly understood. The presence of *Filovirus-like* fragments in *N. lasiurus* and *O. microtis* suggests that these rodents might play a role in maintaining these viruses in nature. However, these findings, along with existing literature, highlight the need for additional studies to better understand the role of these rodents in *Filovirus* ecology and to assess potential transmission risks to other species, including humans. Further collections in the studied areas are essential to obtain more sequences and elucidate the relationship between rodents and these viruses.

## Data Availability

The sequences obtained here was deposited in the GenBank database under accessions PQ336022 (Mammarenavirus sp.), PQ336023 - PQ336025 (Filoviridae sp.), and the raw sequence reads generated are available in the NCBI Sequence Read Archive (SRA) database under BioProject PRJNA1157879. The deposit number of the sequences can be seen in the images.
