# Supplementary material for: Viral diversity in wild rodents in the regions of Canaã de Carajás and Curionopólis, State of Pará, Brazil
Source: Front Microbiol. 2025 Jan 7;15:1502462. doi: 10.3389/fmicb.2024.1502462 (PMC11747277; doi:10.3389/fmicb.2024.1502462)
Supplement: Supplementary file 4 [file Image_1.pdf]

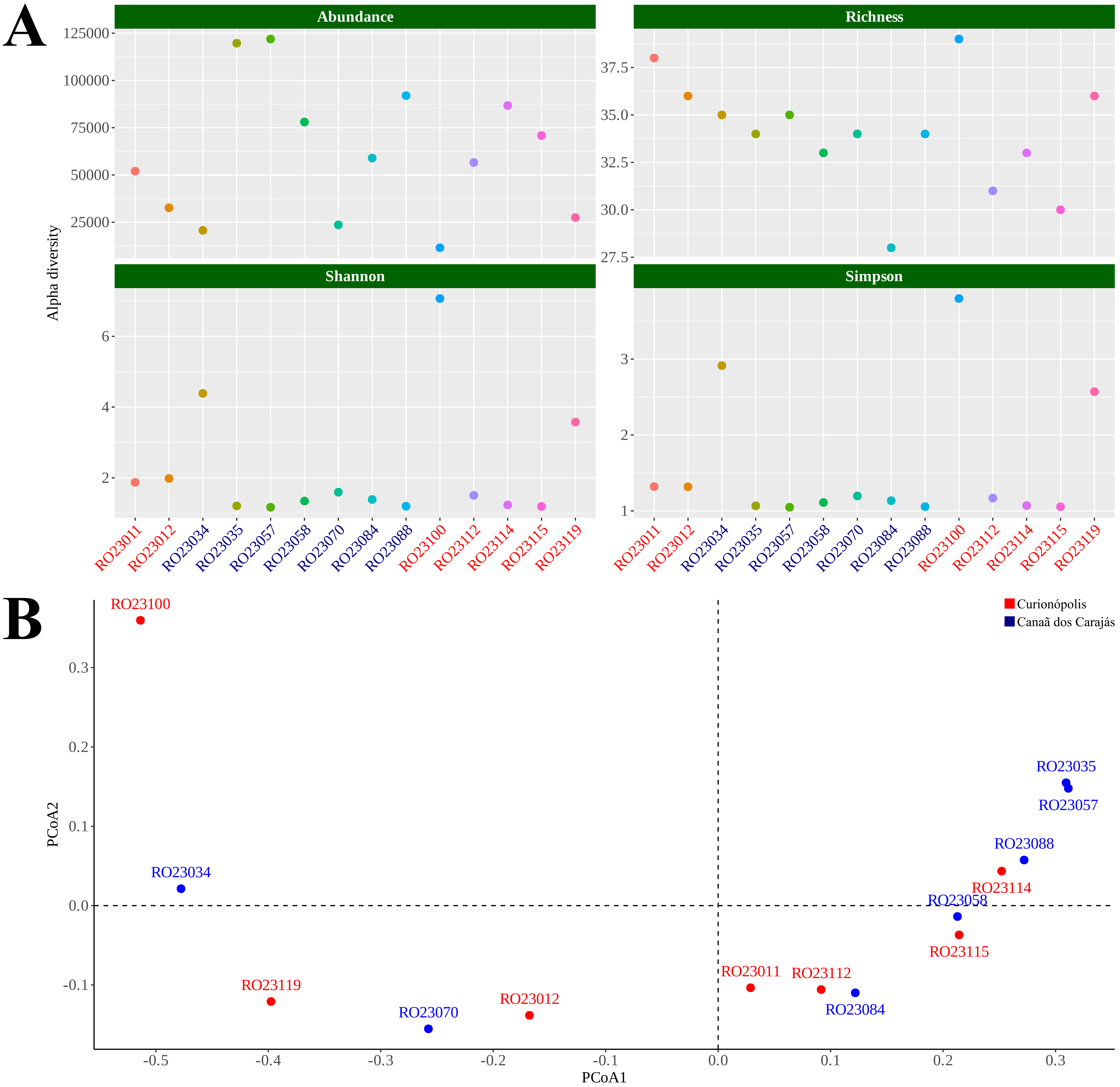

Supplementary Figure 1.(A) Alpha diversity plots showing abundance, richness, and the Shannon and Simpson diversity indices. **(B)** Principal component analysis (PCoA) based on the Bray-Curtis dissimilarity matrix. Samples from the Canaã dos Carajás and Curionópolis regions are highlighted in blue and red, respectively.
