# Supplementary material for: Viral diversity in wild rodents in the regions of Canaã de Carajás and Curionopólis, State of Pará, Brazil
Source: Front Microbiol. 2025 Jan 7;15:1502462. doi: 10.3389/fmicb.2024.1502462 (PMC11747277; doi:10.3389/fmicb.2024.1502462)
Supplement: Supplementary file 5 [file Image_2.pdf]

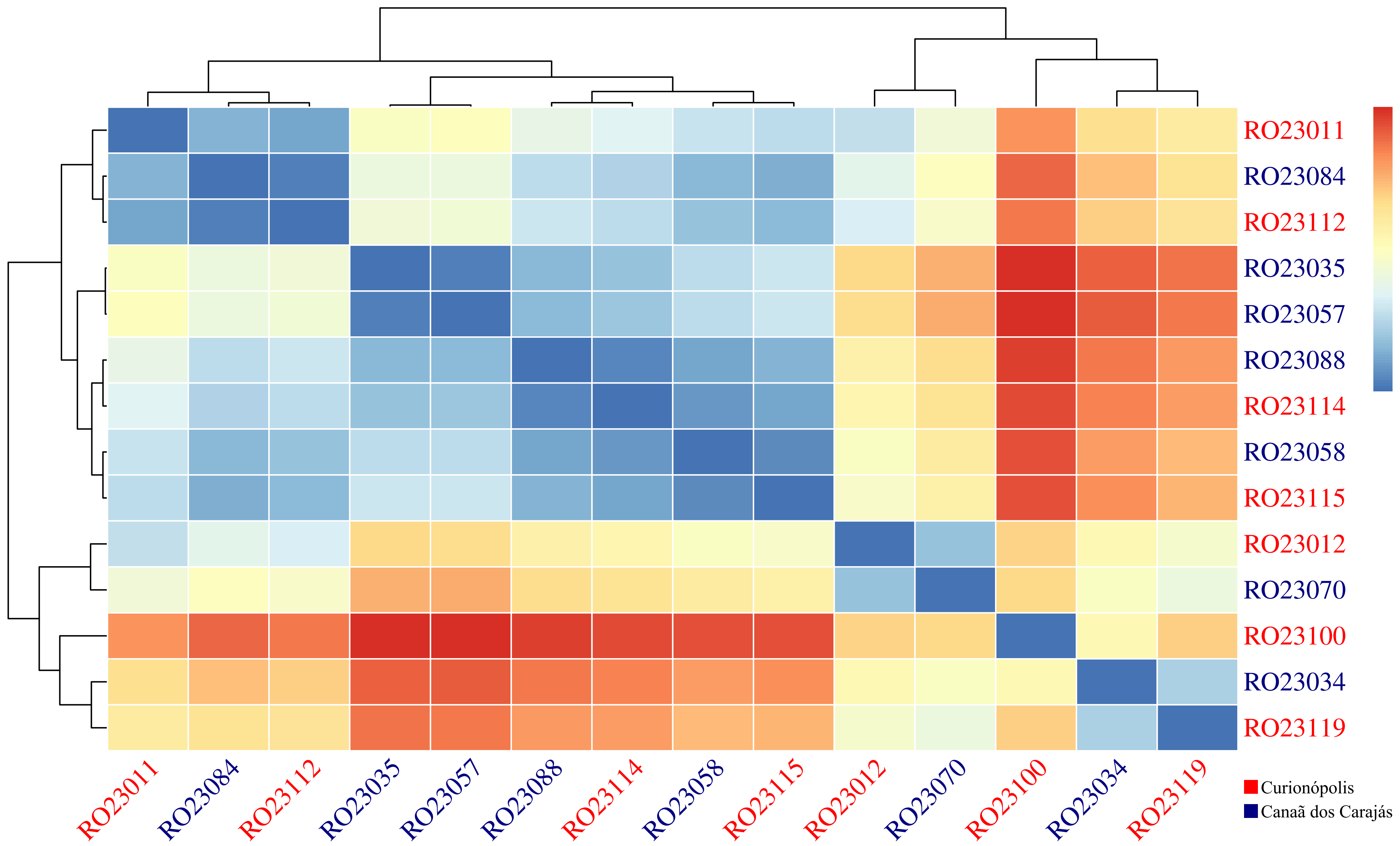

Supplementaty figure 2. Bray-Curtis dissimilarity matrix, based on the comparison of diversity metrics of viral reads found in the evaluated samples.
