# Supplementary material for: Viral diversity in wild rodents in the regions of Canaã de Carajás and Curionopólis, State of Pará, Brazil
Source: Front Microbiol. 2025 Jan 7;15:1502462. doi: 10.3389/fmicb.2024.1502462 (PMC11747277; doi:10.3389/fmicb.2024.1502462)
Supplement: Supplementary file 6 [file Image_3.pdf]

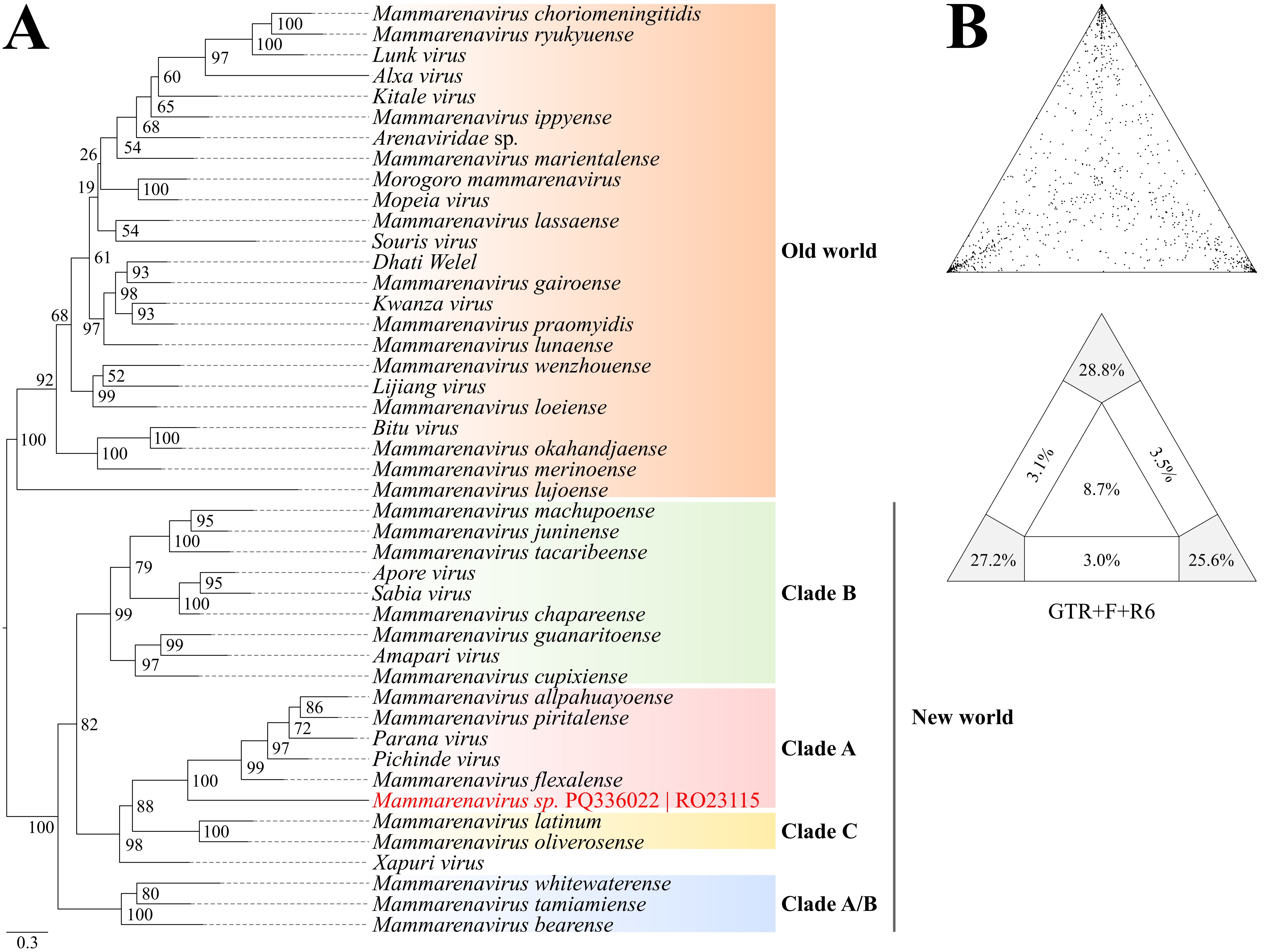

Supplementary Figure 3.(A) Phylogenetic reconstruction using the Maximum Likelihood method based on nucleotide sequences of the glycoprotein region of New and Old World Arenaviruses, including the identified contig highlighted in red. **(B)** Maximum likelihood mapping diagram showing the quality of the phylogenetic signal from the quartet analysis, with 81.6% of quartets resolved.
