# Supplementary material for: Viral diversity in wild rodents in the regions of Canaã de Carajás and Curionopólis, State of Pará, Brazil
Source: Front Microbiol. 2025 Jan 7;15:1502462. doi: 10.3389/fmicb.2024.1502462 (PMC11747277; doi:10.3389/fmicb.2024.1502462)
Supplement: Supplementary file 7 [file Image_4.pdf]

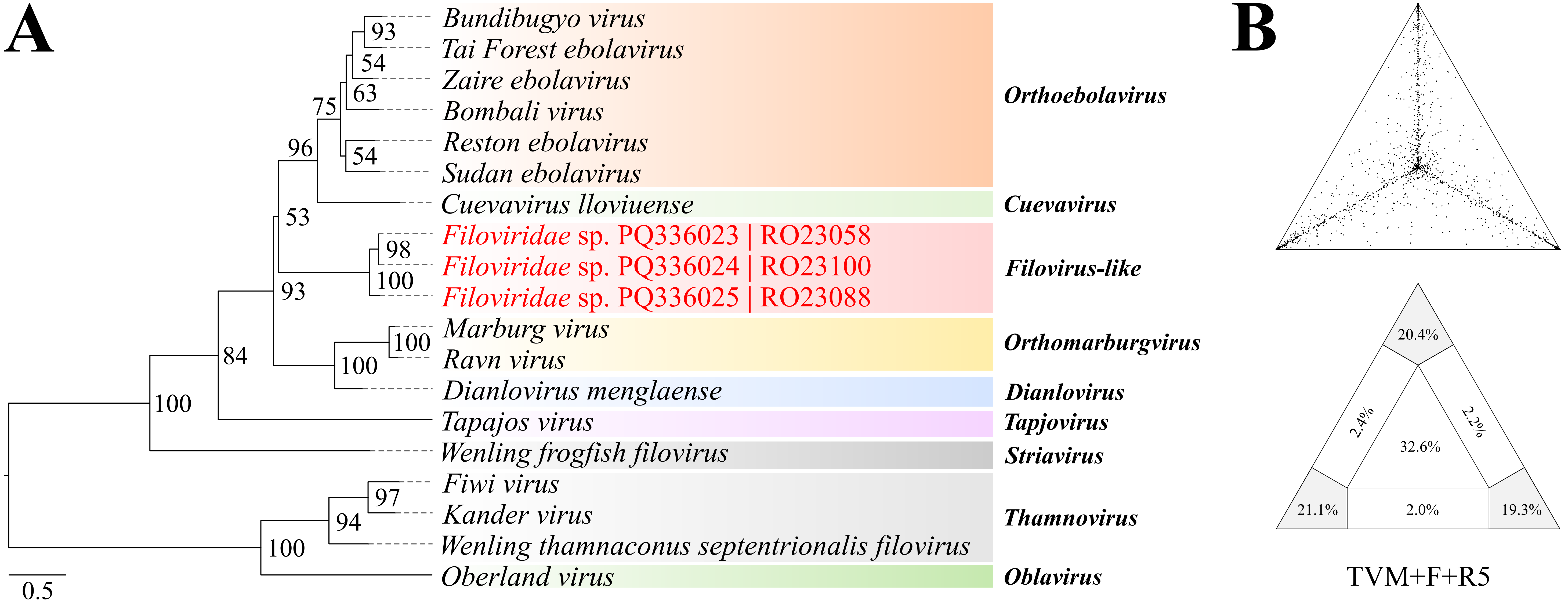

Supplementary Figure 4. (A) Phylogenetic reconstruction using the Maximum Likelihood method based on nucleotide sequences of *Filoviridae*. (B) Maximum Likelihood mapping diagram showing the quality of the phylogenetic signal from the quartet analysis, with 60.8% of quartets resolved
